# Solubilities of CO_2_, O_2_ and N_2_ in rocket propellant 5 under low pressure

**DOI:** 10.1038/s41598-022-08340-8

**Published:** 2022-03-16

**Authors:** Chaoyue Li, Shiyu Feng, Lei Xu, Xiaotian Peng, Weihua Liu

**Affiliations:** 1 School of Mechanical and Electrical Engineering, Jinling Institute of Technology, Nanjing, 211169 China; 2 School of Aerospace Engineering, Nanjing University of Aeronautics and Astronautics, Nanjing, 210016 China

**Keywords:** Energy science and technology, Engineering, Physics

## Abstract

The static method of isochoric saturation was used to measure the solubilities of CO_2_, O_2_ and N_2_ in rocket propellant 5 (RP5) at temperatures ranging from 253.15 to 323.15 K in 10 K intervals and pressures ranging from 0 to 120 kPa. The measurement accuracy of the constructed experimental setup was verified by measuring the solubility of CO_2_ in water. The relative expanded uncertainty (*k* = 2) in the solubility data was less than 4.0%. The solubilities of CO_2_, O_2_ and N_2_ in RP5 increased with pressure. As the temperature increased, the solubility decreased for CO_2_ solubility and increased for O_2_ and N_2_. Henry's constants for the three gases in RP5 decreased over the experimental temperature and pressure ranges in the order of N_2_ > O_2_ > CO_2_. The measured solubilities of CO_2_, O_2_ and N_2_ could be fitted with a modified Krichevsky–Kasarnovsky equation, and the maximum deviation between the measured and calculated data was less than 8.04%, 7.03% and 6.18%, respectively.

## Introduction

Fuel tank combustion explosions are one of the main causes of aircraft safety accidents. The gas mixture of air and fuel vapor in the tank ullage becomes highly combustible in the presence of an external ignition source for oxygen concentrations (volume fractions) above the limiting oxygen concentration (12% for passenger planes and 9% for military aircraft)^1,2^. The results of extensive experiments and calculations have shown that fuel tank inerting is a reasonable and potentially cost-effective approach to reduce fuel tank flammability^3–5^. Fuel tank inerting involves injecting inert gases, such as CO_2_ and N_2_, into a fuel tank to replace the oxygen in ullage, thereby reducing the oxygen concentration below the limiting value. The dissolved gases CO_2_, O_2_ and N_2_ will escape from jet fuel under variations of the ambient pressure and temperature, which has a negative effect on the analysis of fuel tank flammability^6,7^. Knowledge of the solubilities of CO_2_, O_2_ and N_2_ in jet fuel under low pressure is essential for analyzing variations in the oxygen concentration in the ullage. Therefore, it is critical to obtain solubility data to improve the design of aircraft fuel tank inerting systems.

RP5 is a hydrocarbon fuel with a high density, viscosity, heat of combustion, and flash point that is widely used in carrier-based aircraft in China^8^. However, the dissolution characteristics of this fuel depend strongly on the material composition, temperature and pressure, and no universally accurate model is available to predict the gas solubility in RP5 from other known solubility data^9,10^. Barth^11^ measured the solubility of methane in diesel fuel and it was compared to that of methane in pure hexadecane which is similar to diesel fuel with respect to the mean carbon number, and the solubility of methane in diesel fuel is smaller than that of methane in hexadecane. Baird^12^ studied the hydrogen solubility of shale oil and found that the shale oil had a lower hydrogen solubility than most other fuels probably due to the high content of polar phenolic compounds in the oil. Hamme^13^ studied the solubility of neon, nitrogen and argon in distilled water and seawater, and found that the solubility data could be expressed as a polynomial function of temperature and salinity. Thus, experimental tests are necessary to obtain the solubilities of CO_2_, O_2_ and N_2_ in RP5.

Many experimental setups have been developed to measure solubility, such as headspace gas chromatography^14^, absolute gravimetric^15^ and isochoric saturation methods^16,17^. The isochoric saturation method offers the advantages of simple operation, a low experimental cost and high accuracy over other methods and is thus widely used to measure gas solubility in liquids. Liu et al.^18^ measured the solubilities of oxygen, nitrogen and carbon dioxide in JP-10 jet fuel at temperatures ranging from 293 to 343 K and pressures ranging from 0.5 to 7.5 MPa. Jia et al.^19^ investigated the solubilities of carbon dioxide, oxygen and nitrogen in aqueous ethylene glycol solution at temperatures ranging from 263 to 293 K and pressures ranging from 9 to 101 kPa. Shokouhi et al.^20^ experimentally determined the solubility of hydrogen sulfide in aqueous sulfolane solution from 303.15 to 353.15 K and at pressures up to 2 MPa.

An isochoric saturation method was used in this study to measure the solubilities of CO_2_, O_2_ and N_2_ in RP5 at temperatures ranging from 253.15 to 323.15 K and pressures ranging from 0 to 120 kPa. The experimental solubility data could be fitted with a modified Krichevsky–Kasarnovsky equation, and Henry's constant for solvation was calculated at different temperatures.

## Experimental section

### Materials

The CO_2_, O_2_ and N_2_ used in the experiment were purchased from Nanjing Tianze Gas Company with purities above 99.99%. The RP5 was provided by the AVIC Jincheng Nanjing Engineering Institute of Aircraft System with a mass fraction purity of 99%. The RP5 is composed of 78.5% (volume fraction) saturated hydrocarbons, 1.8% unsaturated hydrocarbons and 19.7% aromatic hydrocarbons, that are provided by suppliers. The average molecular mass of RP5 is 155. Information on the experimental materials used in this study is presented in Table 1.

**Table 1:** Information on materials used in the experiment.

Chemical name	Source	Mass fraction purity (%)	CAS number
CO_2_	Nanjing Tianze Gas Company	99.99	124-38-9
O_2_		99.99	7782-44-7
N_2_		99.99	7727-37-9
RP5	AVIC Jincheng Nanjing Engineering Institute of Aircraft System	99	8008-20-6

### Experimental apparatus and method

**Figure 1:**
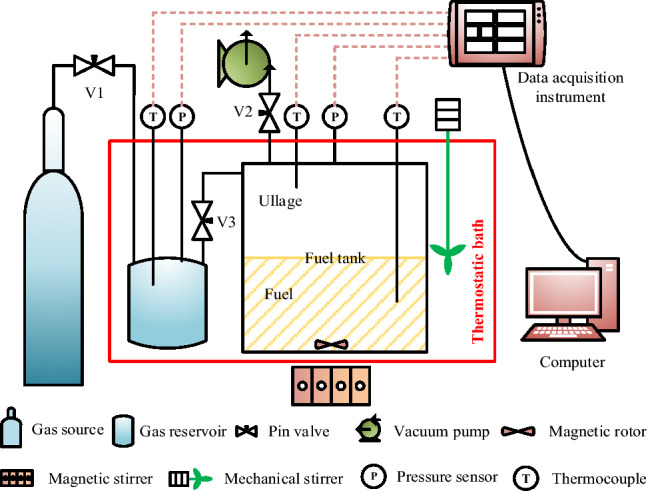
Schematic of experimental system for measuring solubility.

The isochoric saturation method was used to measure the solubilities of CO_2_, O_2_ and N_2_, where the experimental system is presented in Fig. 1. The experimental apparatus consists of a gas source, a gas reservoir, a fuel tank, three pin valves, a vacuum pump, a magnetic rotor, a magnetic stirrer, a mechanical stirrer, a thermostatic bath (FDL BC-3006), three thermocouples (Model K), two pressure sensors (HSTL-800), a data acquisition system and a computer.

The water storage method was used to measure the volumes of the gas reservoir and fuel tank, including the line and valves. Disconnecting the gas reservoir from fuel tank and degassed water is injected into the gas reservoir from valve 1 until the gas reservoir is filled. The volume of gas reservoir could be measured by measuring the volume of water and repeated three times. The same method is applied to measure the volume of the fuel tank. The volumes of the gas reservoir and fuel tank are 332 ± 0.2 mL and 469 ± 0.2 mL, respectively. A thermostatic bath is used to maintain a constant temperature in the fuel tank with an error range of 0.02 K. The test range of the thermocouple is 243.15–373.15 K with the precision of 0.02 K. The test range of the pressure sensor in the gas reservoir is 0–400 kPa with a precision of 0.1 kPa over the full pressure range.

The gas tightness of the experimental system is checked before making measurements by injecting compressed air at 300 kPa into the system; the experimental requirements are met if the pressure drop is less than 1 kPa after 24 h^18^. First, approximately 260 g of RP5 are poured into the fuel tank, and the temperature of the thermostatic bath is set to the experimental temperature. Second, the air in the gas reservoir and fuel tank is degassed by a vacuum pump, and dissolved air escapes from the fuel because of the decrease in the pressure. Third, V1 is opened, V3 is closed, and either CO_2_, O_2_ or N_2_ is loaded into the gas reservoir at the given temperature and pressure. Finally, V3 is opened to transfer gas into the fuel tank, and the pressure decreases as the gas dissolves in the fuel and reaches solution equilibrium. A magnetic stirrer is turned on during the experiment to accelerate the dissolution of CO_2_, O_2_ or N_2_ in RP5 until the temperature and pressure no longer change.

The gas solubility in PR5 is presented as a mole fraction, that is, the ratio of the number of moles of dissolved gas to the total number of moles of gas and fuel. The gas solubility can be expressed as follows:

1 $$x = \frac{{n_{{\text{g,d}}} }}{{n_{{\text{g,d}}} + n_{{\text{l}}} }}$$

where *x* is the mole fraction; *n*_g,d_ is the number of moles of gas dissolved in fuel; and *n*_l_ is the number of moles of fuel.

The fuel mole number is calculated as follows:

2 $$n_{{\text{l}}} = \frac{{m_{{\text{l}}} }}{{M_{{\text{l}}} }}$$

where *m*_l_ is the mass of the fuel, kg; and *M*_l_ is the molecular mass of the fuel.

The mole number of the dissolved gas can be expressed as follows:

3 $$n_{{\text{g,d}}} = \frac{{(\rho_{{\text{g,i}}} - \rho_{{\text{g,f}}} )V_{{\text{G}}} - \rho_{{\text{g,u}}} V_{{\text{u}}} }}{{M_{{\text{g}}} }}$$

where *ρ*_g,i_ and *ρ*_g,f_ are the densities of the gas in the gas reservoir before and after transfer to the fuel tank, respectively, kg/m^3^; *ρ*_g,u_ is the density of gas in the fuel tank ullage after the transfer; and *V*_G_ and *V*_u_ are the volume of the gas reservoir and the fuel tank ullage, respectively, m^3^; *M*_g_ is the molecular mass of gas.

The gas densities *ρ*_g,i_ and *ρ*_g,f_ at a given temperature and pressure can be obtained from REFPROP 9.1^21^. The fuel tank ullage can be written as follows:

4 $$V_{{\text{u}}} = V_{{\text{f}}} - V_{{\text{l}}}$$

where *V*_f_ is the fuel tank volume, m^3^; and *V*_l_ is the volume of the liquid PR5 jet fuel, m^3^.

The fuel volume can be expressed as follows:

5 $$V_{{\text{l}}} = \frac{{m_{{\text{l}}} }}{{\rho_{{\text{l}}} }}$$

where *ρ*_l_ is the density of fuel, kg/m^3^.

**Figure 2:**
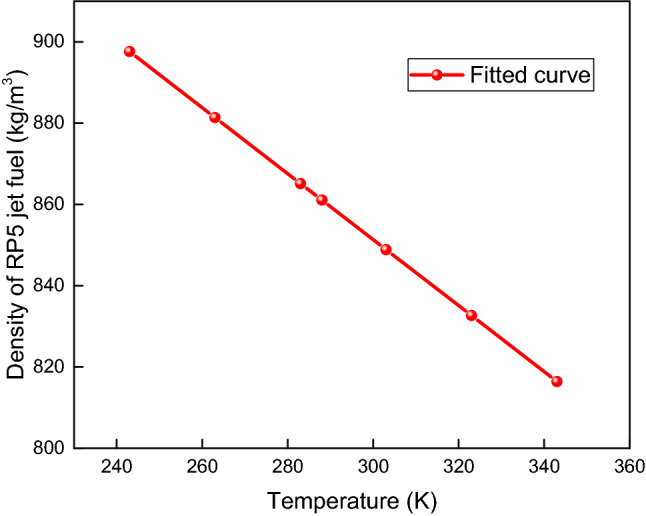
Density of RP5 versus temperature.

The temperature dependence of the RP5 density affects the fuel volume calculation. Therefore, to determine the solubility accurately, the RP5 density was measured using a DA-300API electronic densitometer at temperatures ranging from 243.15 to 343.15 K and atmospheric pressure. The experimental data for the density versus temperature shown in Fig. 2 could be fitted with a linear function as follows:

6 $$\rho_{{\text{l}}} = 1094.92 - 0.81T$$

where *T* is the temperature, K.

The mole fraction *x* of gas dissolved in the fuel can thus be expressed as follows:

7 $$x = \frac{{(\rho_{{\text{g,i}}} - \rho_{{\text{g,f}}} )V_{{\text{G}}} - \rho_{{\text{g,u}}} (V_{{\text{f}}} - V_{{\text{l}}} )}}{{M_{{\text{g}}} n_{{\text{l}}} + V_{{\text{G}}} (\rho_{{\text{g,i}}} - \rho_{{\text{g,f}}} ) - \rho_{{\text{g,u}}} (V_{{\text{f}}} - V_{{\text{l}}} )}}$$

The expanded uncertainty in the solubility mole fraction *U*(*x*) can be expressed as follows^22^:

8 $$U(x) = ku(x) = k\sqrt {\sum {u_{{\text{i}}}^{{2}} } (x)}$$

where *U*(*x*) is the expanded uncertainty in the mole fraction; *k* is the coverage factor that can be considered as 2; *u*(*x*) is the combined standard uncertainty; and *u*_i_(*x*) is the uncertainty in each influencing factor.

Equations (1)–(7) can be combined to express *U*(*x*) as follows:

9 $$U(x) = k\sqrt {\begin{array}{*{20}l} {\left( {\frac{{\updelta x}}{{\updelta V_{{\text{G}}} }}} \right)^{2} u^{2} (V_{{\text{G}}} ) + \left( {\frac{{\updelta x}}{{\updelta \rho_{{\text{g,i}}} }}} \right)^{2} u^{2} (\rho_{{\text{g,i}}} ) + \left( {\frac{{\updelta x}}{{\updelta \rho_{{\text{g,f}}} }}} \right)^{2} u^{2} (\rho_{{\text{g,f}}} )} \hfill \\ {\quad + \left( {\frac{{\updelta x}}{{\updelta \rho_{{\text{g,u}}} }}} \right)^{2} u^{2} (\rho_{{\text{g,u}}} ) + \left( {\frac{{\updelta x}}{{\updelta V_{{\text{f}}} }}} \right)^{2} u^{2} (V_{{\text{f}}} ) + \left( {\frac{{\updelta x}}{{\updelta V_{{\text{l}}} }}} \right)^{2} u^{2} (V_{{\text{l}}} ) + \left( {\frac{{\updelta x}}{{\updelta n_{{\text{l}}} }}} \right)^{2} u^{2} (n_{{\text{l}}} )} \hfill \\ \end{array} }$$

The expanded uncertainties in the measurement variables in the experiment are as follows: temperature (0.023 K), mass of RP5 (0.00002 g), pressure (0.12 kPa), volume of gas reservoir and fuel tank (0.2 mL), density of CO_2_ (0.1%), density of O_2_ (0.06%), and density of N_2_ (0.04%). The relative expanded uncertainty in the experimental solubility data is less than 4.0% when *k* is 2 (In general, the value of the coverage factor *k* is chosen on the basis of the desired level of confidence to be associated with the interval defined by *U* = *kuc*. Typically, *k* is in the range 2–3. When the normal distribution applies and *uc* has negligible uncertainty, *U* = 2*uc* (*k* = 2) defines an interval having a level of confidence of approximately 95%. To be consistent with current international practice, the value of *k* to be used at NIST for calculating *U* is, by convention, *k* = 2^22^.).

### Ethics approval

The research for this article do not include human or animal subjects.

### Verification of accuracy of experimental apparatus

To verify the accuracy of the apparatus for measuring gas solubility in RP5, the solubility of CO_2_ in water was measured using the experimental system at temperatures ranging from 283.15 to 323.15 K and pressures ranging from 30 to 340 kPa; the results are shown in Table 2.

**Table 2:** Solubility (mole fraction) of CO_2_ in water.

*T*/K	*p*/kPa	*x*	U(x)/ × 10^−4^
283.12	259.65	0.0035	1.35
283.16	176.71	0.0015	1.24
293.15	223.56	0.0017	1.19
293.17	156.23	0.0012	1.05
303.18	273.52	0.0016	2.15
303.16	200.35	0.0012	1.98
313.15	321.38	0.0027	3.56
313.14	184.88	0.0017	3.21
323.13	335.12	0.0023	3.05
323.19	196.57	0.0014	2.98

**Figure 3:**
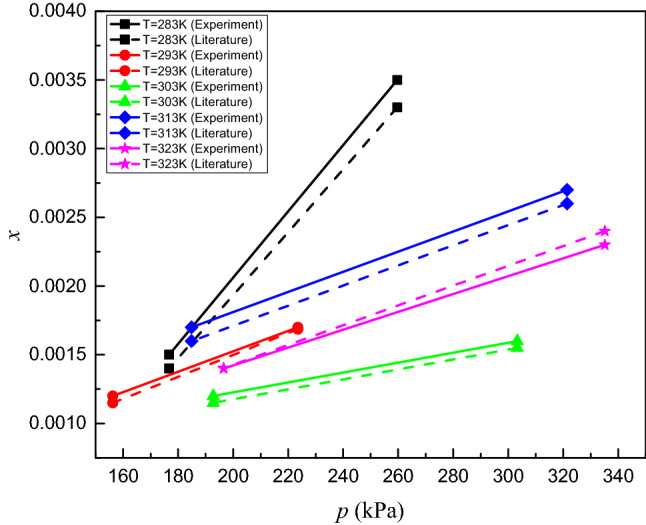
Comparisons of the experimental solubility (mole fraction) of CO_2_ in water with data from the literature.

Figure 3 is a comparison of the experimental data against data obtained from the literature^19^, where the average relative deviation and maximum deviation are 3.89% and 6.81%, respectively. Therefore, the experimentally obtained solubility of CO_2_ in water agrees well with the literature values, and the accuracy of the apparatus meets solubility measurement requirements.

## Results and discussion

### Experimental solubility

**Figure 4:**
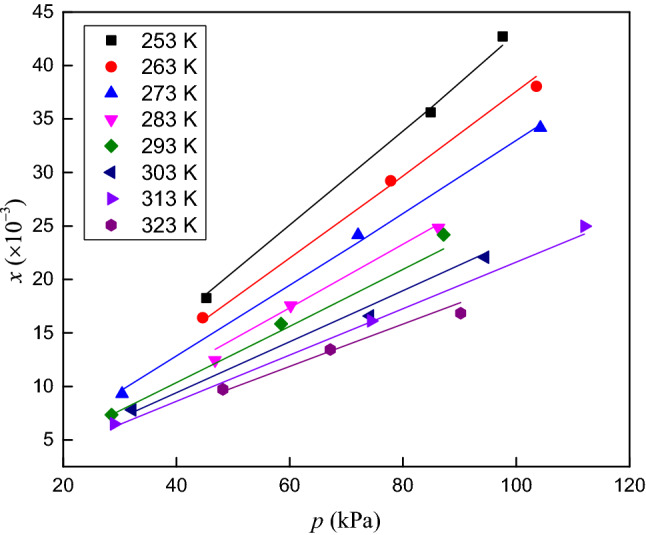
Solubility (mole fraction) of CO_2_ in RP5 versus temperature and pressure.

**Figure 5:**
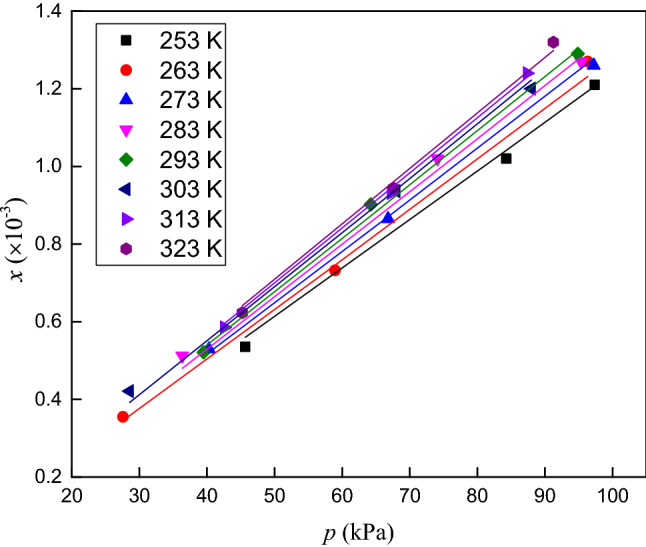
Solubility (mole fraction) of O_2_ in RP5 versus temperature and pressure.

**Figure 6:**
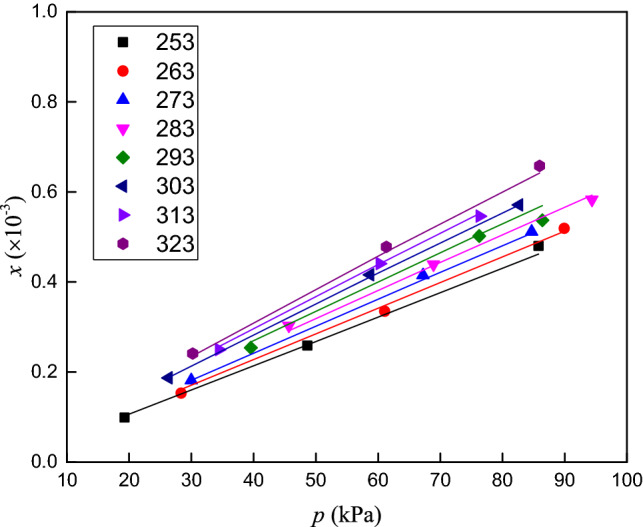
Solubility (mole fraction) of N_2_ in RP5 versus temperature and pressure.

The solubilities of CO_2_, O_2_ and N_2_ in RP5 were measured at temperatures ranging from 253.15 to 323.15 K and pressures ranging from 0 to 120 kPa. The experimental data and the expanded uncertainties in the mole fraction are listed in Tables 3, 4 and 5. The solubility data versus temperature and pressure are presented in Figs. 4, 5 and 6.

**Table 3:** Solubility (mole fraction) and associated uncertainty of CO_2_ in RP5.

*T*/K	*p*/kPa	*x*/ × 10^−3^	*U*(*x*)/ × 10^−3^	*T*/K	*p*/kPa	*x*/ × 10^−3^	*U*(*x*)/ × 10^−3^
253.15	45.287	18.26	0.035	293.25	28.550	7.35	0.062
253.25	84.922	35.61	0.042	293.15	58.493	15.85	0.068
253.15	97.628	42.71	0.047	293.15	87.195	24.17	0.075
263.15	44.659	16.41	0.058	303.15	32.208	7.81	0.055
263.15	77.842	29.22	0.062	303.15	74.180	16.58	0.062
263.25	103.574	38.05	0.069	303.15	94.527	22.07	0.071
273.15	30.408	9.31	0.049	313.15	28.991	6.49	0.061
273.15	72.079	24.16	0.063	313.15	74.385	16.14	0.063
273.25	104.280	34.18	0.071	313.15	112.058	24.97	0.073
283.15	46.807	12.45	0.056	323.35	48.205	9.73	0.046
283.15	60.184	17.56	0.074	323.15	67.185	13.44	0.051
283.25	86.276	24.82	0.079	323.15	90.207	16.83	0.058

**Table 4:** Solubility (mole fraction) and associated uncertainty of O_2_ in RP5.

*T*/K	*p*/kPa	*x*/ × 10^−3^	*U*(*x*)/ × 10^−3^	*T*/K	*p*/kPa	*x*/ × 10^−3^	*U*(*x*)/ × 10^−3^
253.45	45.667	0.54	0.0048	293.35	39.499	0.52	0.0041
253.15	84.285	1.02	0.0051	293.15	64.228	0.90	0.0052
253.15	97.374	1.21	0.0069	293.15	94.877	1.29	0.0062
263.05	27.550	0.36	0.0044	303.15	28.540	0.42	0.0066
263.15	58.944	0.73	0.0053	303.15	67.991	0.94	0.0073
263.15	96.334	1.27	0.0065	303.25	87.997	1.20	0.0078
273.25	40.281	0.53	0.0032	313.15	42.556	0.59	0.0047
273.35	66.810	0.87	0.0054	313.35	67.220	0.93	0.0058
273.15	97.224	1.26	0.0065	313.15	87.366	1.24	0.0078
283.25	36.331	0.51	0.0058	323.15	45.230	0.62	0.0033
283.35	74.112	1.02	0.0071	323.25	67.589	0.94	0.0048
283.15	95.352	1.27	0.0071	323.15	91.254	1.32	0.0055

**Table 5:** Solubility (mole fraction) and associated uncertainty of N_2_ in RP5.

*T*/K	*p*/kPa	*x*/ × 10^−3^	*U*(*x*)/ × 10^−3^	*T*/K	*p*/kPa	*x*/ × 10^−3^	*U*(*x*)/ × 10^−3^
253.35	19.250	0.099	0.0031	293.25	39.550	0.98	0.0025
253.15	48.633	0.259	0.0044	293.25	76.267	1.93	0.0050
253.25	85.799	0.480	0.0053	293.15	86.411	2.06	0.0053
263.15	28.336	0.153	0.0070	303.15	26.331	0.72	0.0018
263.15	61.083	0.335	0.0079	303.25	58.740	1.60	0.0041
263.35	89.926	0.519	0.0091	303.15	82.669	2.19	0.0057
273.15	29.994	0.182	0.0052	313.35	34.441	0.96	0.0025
273.25	67.240	0.415	0.0068	313.15	60.285	1.69	0.0044
273.15	84.685	0.512	0.0079	313.25	76.338	2.10	0.0054
283.15	45.662	0.303	0.0011	323.35	30.204	0.91	0.0024
283.15	68.917	0.439	0.0018	323.15	61.349	1.84	0.0047
283.25	94.385	0.583	0.0023	323.15	85.958	2.52	0.0065

**Figure 7:**
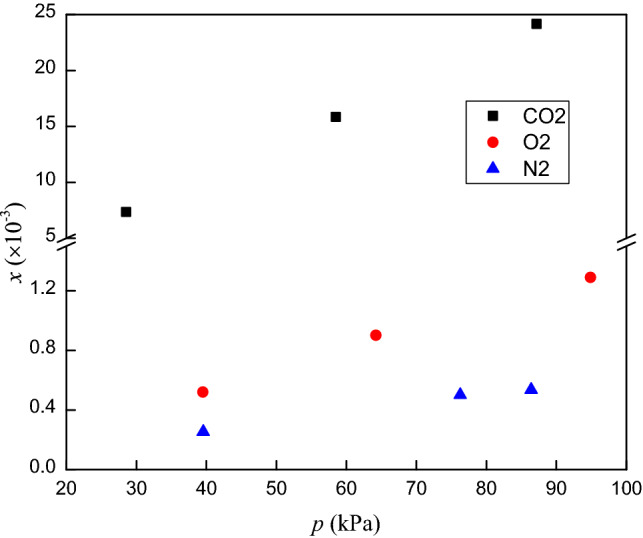
Solubility (mole fraction) of CO_2_, O_2_ and N_2_ in RP5 at 293.15 K.

The solubilities of the three gases in RP5 clearly increase with pressure. The mole fraction of CO_2_ in RP5 decreases with increasing temperature. By contrast, the mole fractions of O_2_ and N_2_ in RP5 increase with temperature. Figure 7 shows the solubilities of CO_2_, O_2_ and N_2_ in RP5 at 293.15 K, where the gas solubility decreases in the order CO_2_ > O_2_ > N_2_ at the same temperature and pressure. The solubility of CO_2_ in RP5 increase faster than those of O_2_ and N_2_ as pressure increase, which indicates the solubility of CO_2_ in RP5 is more sensitive to pressure.

### Solubility data analysis

Henry's law is the most commonly used correlation for evaluating the solubility of a gas dissolved in a liquid solvent. A more general form of Henry's law that accounts for pressure effects is based on a thermodynamic correlation known as the Krichevsky–Kasarnovsky equation^9,23,24^ and can be expressed as follows:

10 $$\ln \frac{f(T,p)}{x} = \ln H + \frac{{V_{1}^{\infty } (p - p_{2}^{s} )}}{RT}$$

where *f*(*T*,*p*) is the gas fugacity at the given temperature and pressure, MPa; *H* is Henry's constant, MPa; $V_{1}^{\infty }$ is the partial molar volume of the gas in the respective solvent, L/mol; $p_{2}^{s}$ is the saturated vapor pressure of the solvent, MPa; and *R* is the gas constant, 8.314 J/(mol K). The gas fugacity can be obtained using REFPROP 9.1 software^21^. The $p_{2}^{s}$ term can neglected over the very low temperature range used in the experiment.

Henry's constant and $V_{1}^{\infty }$ can both be expressed as functions of the temperature as follows:

11 $$\ln H = A + \frac{B}{T}$$

12 $$V_{1}^{\infty } = a + bT + cT^{2}$$

where A, B, a, b, and c are adjustable parameters.

The modified Krichevsky–Kasarnovsky equation can be expressed as follows:

13 $$\ln \frac{f}{x} = A + \frac{B}{T} + \frac{{(a + bT + cT^{2} )p}}{RT}$$

**Figure 8:**
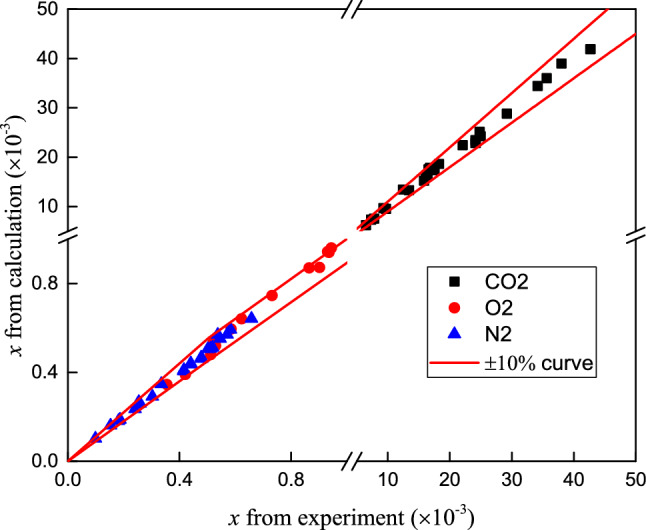
Deviation between experimental data (mole fraction) and value calculated using Eq. (13).

Equation (13) can be used to obtain correlations for the individual solubilities of the three gases in RP5. Table 6 presents the adjustable parameters obtained by fitting the experimental data. Figure 8 shows the deviation between the experimental data and the value calculated using Eq. (13).

**Table 6:** Parameters obtained by fitting experimental data.

Parameters	CO_2_	O_2_	N_2_
A/MPa	4.134	3.699	3.349
B/MPa	− 812.309	180.814	478.239
a/J mol^−1^	− 12,427.616	21,008.713	− 15,786.025
b/J mol^−1^ K^−1^	54.231	− 155.212	80.847
c/J mol^−1^ K^−2^	− 0.050	0.277	− 0.079

The deviation between the experimental data and the calculated values is less than 10%. The absolute average deviations (AADs) and maximum deviations (MDs) are determined to analyze the accuracy of the solubility calculated by the modified KK equation. The AAD and MD are expressed below:

14 $${\text{AAD}} = \frac{{\sum\nolimits_{i}^{N} {\left| {\frac{{x_{\exp } - x_{cal} }}{{x_{\exp } }}} \right|} }}{N} \times 100\%$$

15 $${\text{MD}} = \max \left( {\frac{{x_{\exp } - x_{cal} }}{{x_{\exp } }} \times 100\% } \right)$$

where *x*_exp_ and *x*_cal_ are the experimental and calculated mole fractions of gas in RP5, respectively, and N is the number of experimental data points.

**Figure 9:**
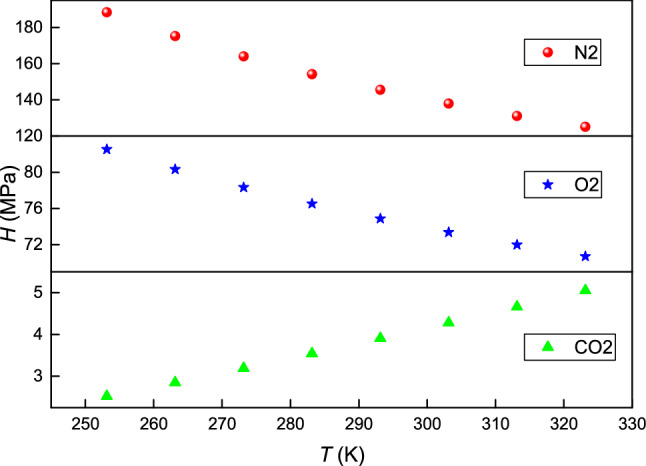
Henry's constant versus temperature for CO_2_, O_2_ and N_2_ in RP5.

The AADs for CO_2_, O_2_ and N_2_ are 2.74%, 2.25% and 2.17%, respectively. The MD values for CO_2_, O_2_ and N_2_ are 8.04%, 7.03% and 6.18%, respectively. Table 7 and Fig. 9 show the values of Henry's constant calculated using Eq. (11) for CO_2_, O_2_ and N_2_. Henry's constant decreases as the temperature increases for O_2_ and N_2_ but increases with the temperature for CO_2_, that similar to the trend of CO_2_, O_2_ and N_2_ solubility in JP-10 in literature^18^. Henry's constant for the three gases in RP5 decreases in the order N_2_ > O_2_ > CO_2_, which is opposite to the trend observed for the solubility.

**Table 7:** Henry's constant for CO_2_, O_2_ and N_2_ in RP5.

T/K	H/MPa		
	CO_2_	O_2_	N_2_
253.15	2.52	82.54	188.32
263.15	2.85	80.33	175.28
273.15	3.19	78.33	163.99
283.15	3.54	76.52	154.16
293.15	3.91	74.87	145.53
303.15	4.28	73.37	137.91
313.15	4.66	71.98	131.13
323.15	5.05	70.71	125.08

## Conclusions

The isochoric saturation method was used to measure the solubilities of CO_2_, O_2_ and N_2_ in RP5 at temperatures ranging from 253.15 to 323.15 K and pressures ranging from 0 to 120 kPa. The solubility, as represented by the gas mole fraction, decreases with increasing temperature for CO_2_ and increases with the temperature for O_2_ and N_2_. The solubilities for the three gases decrease in the order CO_2_ > O_2_ > N_2_ at the same temperature and pressure. The solubilities calculated using the modified KK equation are in good agreement with the experimental data. The absolute average deviations for CO_2_, O_2_ and N_2_ are 2.74%, 2.25% and 2.17%, respectively. Henry's constant increases with the temperature for CO_2_ and decreases with increasing temperature for O_2_ and N_2_, which represents an opposite trend to that observed for the solubility. Henry's constant for the three gases decreases in the order N_2_ > O_2_ > CO_2_ at the same temperature.

## Data Availability

All data generated or analysed during this study are included in this published article.
